# Dietary nitrate supplementation does not improve resistance exercise performance in resistance-trained women

**DOI:** 10.1007/s00421-026-06206-9

**Published:** 2026-04-03

**Authors:** Sydney N. Brennan, Justin M. Foster, Raymond T. Gerardo, Trevor J. Molnar, Ryan Tran, Christopher B. Sottile, Kyle S. Geppert, Michael Egiazarian, Abigail F. Ballhagen, Money Ghimire, John R. M. Renwick, Lewis A. Gough, Stewart D. Gonzalez, Jason D. Allen, Samantha N. Rowland, Stephen J. Bailey, Adam Pennell, Rachel Tan

**Affiliations:** 1 Department of Sports Medicine, Pepperdine University, Malibu, CA USA; 2 Department of Kinesiology, School of Education and Human Development, University of Virginia, Charlottesville, VA USA; 3 Department of Kinesiology, McMaster University, Hamilton, ON Canada; 4 Research for Human Performance and Health Laboratory, Centre for Life and Sport Science, Birmingham City University, Birmingham, UK; 5 Pepperdine Sports Performance, Pepperdine University, Malibu, CA USA; 6 Division of Cardiovascular Medicine, School of Medicine, University of Virginia Health System, Charlottesville, VA USA; 7 School of Sport, Exercise and Health Sciences, Loughborough University, Loughborough, UK; 8 Natural Sciences Division, Pepperdine University, 24255 Pacific Coast Highway, Malibu, 90263 USA

**Keywords:** Nitric oxide, Beetroot, Muscle, Weightlifting, Ergogenic aid, Training, Fatigue, Females

## Abstract

Dietary nitrate supplementation has been purported to enhance explosive exercise performance; however, research examining the efficacy of nitrate to improve resistance exercise performance, such as weightlifting, in women is scarce. The purpose of this study was to investigate the impact of an acute nitrate dose on neuromuscular performance during barbell back squat and bench press. Eighteen resistance-trained women were assigned in a randomized, double-blinded, crossover design to consume: (1) nitrate-depleted beetroot juice (PL; negligible nitrate) and (2) nitrate-rich beetroot juice (BR; ~ 6.7 mmol nitrate) 2.5 h prior to exercise. Participants performed explosive efforts during 1 set of 3 repetitions at 55%1RM, 60%1RM, and 65%1RM, with each set interspersed by 2 min of passive recovery. A linear transducer was used to assess peak and mean power and velocity of each set. There were no differences between conditions for back squat and bench press variables (*P* > 0.05) with the exception that performance declined in BR compared to PL in 60%1RM bench press mean power (BR: 201 ± 61 W vs. PL: 206 ± 61 W; *P* = 0.047, *g*_*z*_ = -0.48) and mean velocity (BR: 0.68 ± 0.08 m/s vs. PL: 0.70 ± 0.08 m/s; *P* = 0.049, *g*_*z*_ = -0.50). These results indicate that acute nitrate ingestion does not improve back squat performance and more research is required to elucidate if nitrate elicits ergolytic effects in resistance-trained women.

**Supplementary Information:**

The online version contains supplementary material available at 10.1007/s00421-026-06206-9.

## Introduction

Dietary nitrate supplementation is a precursor for nitric oxide, an important molecule involved in regulating a plethora of physiological functions (Stamler and Meissner 2001), via the conversion of nitrate to nitrite and then nitrite to nitric oxide (Lundberg et al. 2008). Initially, dietary nitrate was reported to enhance performance during continuous submaximal endurance exercise (Senefeld et al. 2020). Since then, evidence also supports that dietary nitrate supplementation has potential ergogenic effects on explosive, short-duration, high-intensity exercise requiring high-velocity and high-power contractions (Alsharif et al. 2023; Esen et al. 2022; Tan et al. 2023b). Given that the reduction of nitrite to nitric oxide is facilitated in acidic and hypoxic milieu (Castello et al. 2006; Modin et al. 2001), and that these physiological conditions are better reflected in contracting type II compared to type I muscle fibers, this further highlights the ergogenic potential for nitrate to improve physiological responses in type II muscle, which are heavily recruited in resistance exercises such as weightlifting (Morton et al. 2019).

In the few available studies examining the effects of dietary nitrate on weightlifting performance, nitrate has been shown to be effective (Jurado-Castro et al. 2022; Ranchal-Sanchez et al. 2020; Rodríguez-Fernández et al. 2021) and ineffective at improving power output and velocity during back squats (Tan et al. 2022, 2023c, 2025). Consequently, it is currently unclear whether power and velocity during lower-body weightlifting can be improved by nitrate supplementation. While it is possible that the ergogenic potential of nitrate is greater in upper body exercises due to greater proportions of type II muscle fibers in some upper body compared to lower body skeletal muscles (Zinner et al. 2016), the effects of nitrate on power output during bench press exercise are also conflicting. Indeed, there is evidence of improved (e.g., + 19%; Williams et al. 2020) and no effects (Tan et al. 2022, 2025) on bench press power following nitrate supplementation.

Importantly, of the nine studies that have examined the ergogenic potential of dietary nitrate on power and velocity during weightlifting exercise (Jurado-Castro et al. 2022; Montalvo-Alonso et al. 2025; Ranchal-Sanchez et al. 2020; Rodríguez-Fernández et al. 2021; Tan et al. 2022, 2023b, c, 2025; Williams et al. 2020), to our knowledge, only one study has exclusively studied women (Jurado-Castro et al. 2022). In this study, nitrate supplementation enhanced power and velocity during 50%1RM Smith-machine back squats and countermovement jump height (Jurado-Castro et al. 2022). The dearth of studies in women is potentially important because the efficacy of nitrate supplementation may differ depending on biological sex due to sex-differences in fiber type composition (Nuzzo 2024), nitrate storage (Dao and Kazin 2007; Fujii et al. 2023; Janssen et al. 2000; Wylie et al. 2019), nitrate metabolism (Hickner et al. 2006), and nitrate reduction capacity (Inoue et al. 2006; Kapil et al. 2018). Indeed, the majority of the limited current evidence in women-only studies suggests that there are no effects of nitrate supplementation in single or repeated sprints (López-Samanes et al. 2022a, b, 2023), strength (López-Samanes et al. 2022a, b, 2023), endurance (Ortiz de Zevallos et al. 2023), power (Ortiz de Zevallos et al. 2024; Poredoš et al. 2022), and economy (Forbes and Spriet 2021; López-Samanes et al. 2023; Ortiz de Zevallos et al. 2023; Poredoš et al. 2022) and that it could be ergolytic for cycling performance (Hogwood et al. 2023, 2024a, b). These data are in contrast to the findings of Jurado-Castro et al., where nitrate improved resistance exercise performance (Jurado-Castro et al. 2022). Since dietary nitrate supplementation may be an ergogenic aid for men (Senefeld et al. 2020), the current limited equivocal evidence and potential *ergolytic* effects in women-only cohorts highlights a critical gap in knowledge that needs to be resolved.

The purpose of this study was to investigate the effects of acute dietary nitrate, provided as concentrated nitrate-rich beetroot juice, on power output and velocity during barbell back squats and bench press in resistance-trained women. We hypothesized that dietary nitrate supplementation would enhance performance variables during back squats and bench press when compared to a nitrate-depleted placebo.

## Materials and methods

### Participants

Twenty resistance-trained women volunteered to participate in this randomized, double-blind, placebo-controlled crossover trial. While a minimum sample size of *n* = 17 was selected using power calculations based on a published report (Williams et al. 2020) (i.e., power of 0.95, an alpha of 0.05, and a ‘large’ Cohen’s *d* effect size threshold of 0.80), we recruited additional participants to account for participant drop out. This sample size falls in line with previous/related investigations that observed significant positive effects of nitrate on weightlifting exercise performance (Rodríguez-Fernández et al. 2021). Resistance-trained was defined as having performed barbell back or front squat and bench press 2–3 times per week, for the past 2 years. Participants were instructed to maintain their normal training regimen throughout the experiment. Participant exclusion criteria were: individuals with contraindications to exercise, cardiometabolic disease, men, women on hormonal contraceptives, women with menstrual cycle irregularities (e.g., amenorrhea, endometriosis, etc.), smokers, and individuals currently consuming dietary supplements containing caffeine, sodium bicarbonate, creatine, beta-alanine, and/or nitric oxide precursor supplements (i.e., nitrate, arginine, citrulline, antioxidants). The experimental protocol, risks and benefits were explained to each participant prior to obtaining informed consent. Participants were assigned an anonymous randomization code. This study was approved by the Institutional Research Ethics Committee (Protocol 24–04-2402 on 2024 May 02) and conformed to the code of ethics of the Declaration of Helsinki. This study was pre-registered to the Open Science Framework database on 2024 May 10 (osf.io/u928b), and was conducted between May 2024 to October 2025 at Pepperdine University, Malibu, United States of America. Two participants dropped out prior to the last experimental visit as they no longer met the inclusion criteria: one was removed due to a change in health status; one experienced secondary amenorrhea. Therefore, data were collected, analyzed and reported for *n* = 18 (mean SD: age 20 ± 3 y, body mass 61 ± 10 kg, height 1.62 ± 0.06 m).

### Experimental overview

Participants reported to the laboratory for a total of 4 × 50 min sessions over an ~ 8 to 10-wk period during the early follicular phase of the menstrual cycle (day 1 to 5 of menses as determined by self-cycle reporting). This phase is generally characterized by more stable and lower levels of ovarian hormones, particularly estrogen––which can increase nitric oxide bioavailability––thereby minimizing potential confounding effects on nitric oxide (Baranauskas et al. 2022; Elliott-Sale et al. 2025). On visit 1, participants underwent informed consent, participant screening, and standardized one repetition maximum (1RM) testing procedures for the determination of exercise intensity for subsequent visits. On visit 2, participants performed familiarization to the experimental protocol and coaching to ensure correct lifting techniques for our protocol which emphasized explosive performance. Then, in a randomized, double-blinded, crossover design, participants were assigned to two experimental conditions using a web-based randomizer (random.org) to receive: 1) placebo consisting of nitrate-depleted beetroot juice (PL; 1 × 70 mL per day containing negligible nitrate) and 2) nitrate-rich beetroot juice (BR; 1 × 70 mL containing 6.7 mmol of nitrate). The same web-based randomizer was used to create a randomization sequence determining the order of exercise between bench press and back squats. Since plasma [nitrate] and [nitrite] return to baseline levels 24-h post-ingestion (Wylie et al. 2013), each condition was separated by a washout period of at least 2 days to ensure that the previous condition or exercise did not impact the subsequent condition. Together, the randomized balance sequence and washout period minimized possible carryover effects. All supplements were identical in taste, smell, and appearance. The supplementation dose was based on previous meta-analyses which demonstrated that > 5 mmol of nitrate (i.e., ingestion of 1 × 70 mL nitrate-rich beetroot ‘shot’ containing ~ 6–6.5 mmol of nitrate) (Shannon et al. 2022) ingested 2 to 3-h prior to exercise is required for performance effects (Senefeld et al. 2020). Participants recorded diet, hydration, sleep and physical activity habits 24-h prior to the first supplemented visit (i.e., visit 3) and were asked to repeat their recorded diet and physical activity log 24-h prior to the subsequent visit (i.e., visit 4) to control for lifestyle factors, and was verified during experimental visits. Participants used period tracking applications on their phones (i.e., Flo, P.Tracker) to monitor and predict menstrual cycle and reported the onset menses via text to schedule experimental visits. All tests were performed at the same time of day. Participants were instructed to: (1) avoid antibacterial mouthwash for the duration of the study since mouthwash interferes with nitrate metabolism (Govoni et al. 2008); and (2) avoid caffeine 8-h, exercise 24-h, and alcohol 24-h before each experimental visit. If participants were regular consumers of non-excluded supplements (e.g., protein powder, multivitamins, etc.) they were required to continue their typical regimen, and were not permitted to initiate a new brand or new supplement regimen during the study.

### Exercise protocol

Participants performed a one-repetition maximum (1RM) test for barbell back squat and barbell bench press as previously described (Tan et al. 2025) and based on *Essentials of Strength Training and Conditioning* (Haff & Triplett, 2016). Briefly, participants completed 5 back squats at 50% of their perceived 1RM, followed by 3 repetitions at 70% of their perceived 1RM with each set interspersed by 2 min of recovery. Subsequently, the load was increased in stepwise increments (0.2 kg to 9 kg) until the participant’s maximum was successfully lifted within 3 to 5 attempts, with each attempt interspersed by 3 min of recovery. This process was then repeated for the determination of bench press 1RM. All participants were required to use standardized procedures for the back squat (i.e., medium grip, parallel depth, neutral stance and spine, lower-body extension to original standing position), and bench press (i.e., medium grip, bar to chest, full extension of arms) throughout the entire duration of the study and were provided coaching cues to ensure standardized technique.

During visit 2, participants performed a familiarization to the exercise protocol to ensure correct lifting technique and to minimize any potential learning effects. Participants completed a warm-up for their randomly selected first resistance exercise (i.e., squat or bench press). Following warm-up, participants performed an explosive lift, using an eccentric–pause–concentric–pause tempo of 1–0–1–2 (each phase in seconds) to emphasize explosive movements and to standardize lifting across participants (Wilk et al. 2018), and using the barbell only (20 kg), for a total of 10 repetitions and 1 set × 5 repetitions at 40%1RM. Then, participants were familiarized to the experimental protocol consisting of 1 set × 3 repetitions at 55%1RM, 1 set × 3 repetitions at 60%1RM, and 1 set × 3 repetitions at 65%1RM, with 2 min of recovery between each set. The same protocol was performed for the remaining resistance exercise following a warm-up specific to that exercise (e.g., if squats were performed first, bench was performed second, and vice versa). Standardized coaching techniques were provided during this session.

During the experimental visits (i.e., visits 3 and 4), participants reported to the laboratory to perform the experimental protocol for the determination of primary outcomes of muscular power and velocity––as familiarized with on visit 2––at 2.5 h post-supplementation. The movement tempo of individual movement phases during weightlifting exercise was controlled for using an eccentric–pause–concentric–pause tempo of 1–0–1–2 (each phase in seconds) to emphasize explosive movements and to standardize lifting across participants (e.g., descend quickly, ascend quickly, brief pause at the top, and repeat) (Wilk et al. 2018). During these visits, participants completed back squats and bench press in a randomized order that was consistent within participants and across conditions. Participants completed a standardized warm up for back squats and bench press as described in the previous section. Following this, a linear position transducer (GymAware Power Tool, Kinetic Performance Technology, Mitchell, Australia) was attached to the barbell to assess power and velocity of movement. Power and velocity were determined during the experimental protocol consisting of 1 set × 3 repetitions at 55% 1RM, 1 set × 3 repetitions at 60% 1RM, and 1 set × 3 repetitions at 65% 1RM, with each set interspersed by 2 min of recovery. For both resistance exercises, participants were instructed to lift the weight as fast as possible to produce an explosive movement. Standardized encouragement and technical feedback were given to participants during all sets.

To maximize the applicability and implementation of the study results to current practices in strength and conditioning, this protocol follows current research- and National Strength and Conditioning Association (NSCA) supported guidelines (e.g., < 6 reps; 30–70%1RM, 2–5 min rest, etc.) for power training (Cormie et al. 2007; Haff & Triplett, 2016; McGuigan & National Strength and Conditioning Association (U.S.), 2017; Soriano et al. 2017). Moreover, to maximize translation of study results to current training for sports teams, the exercise protocol was verified by the current strength and conditioning specialists for our National Collegiate Athletics Association (NCAA) Division I baseball team as current and relevant for their programming, which is also based on research- and/or evidence-based practices (Baker et al. 2001; Davies et al. 2020; Haff & Triplett, 2016; Verkhoshansky & Siff 2009) as well as grounded within the NSCA Professional Standards and Guidelines (2017).

### Supplementation procedures

In a randomized, double-blinded, crossover design, participants were assigned to two supplementation periods to consume (1) nitrate-depleted beetroot juice (PL; negligible nitrate per 70 mL; Beet It; James White Drinks Ltd.; Ipswich, UK) and (2) nitrate-rich beetroot juice (BR; ~ 6.7 mmol of nitrate per 70 mL; Beet It; James White Drinks Ltd.; Ipswich, UK). During each condition, participants consumed a carbohydrate snack of their choice, which was recorded and replicated with each visit, along with 1 × 70 mL of their allocated supplement 2.5-h prior to exercise to align the attainment of peak nitric oxide bioavailability (i.e., plasma [nitrite]) with the start of exercise (Wylie et al. 2013). During this 2.5-h time period between supplementation and the start of exercise, participants did not ingest anything other than the ad libitum consumption of water and this fasting time was consistently replicated for each supplemented visit. Consumption of supplements was verified via text prior to arriving at the laboratory, log records, and verbal confirmation and recorded on a questionnaire upon arriving to the laboratory. Empty supplement bottles were returned to researchers wherein blinded manufacturing codes were recorded. The effectiveness of the blinding procedures was recorded and assessed by asking whether the participants noticed any difference in the supplements ingested via verbal questions and completing a form at the start and end of the experimental visits, respectively.

### Measurements

#### Plasma nitrate and nitrite analysis

A resting venous blood sample was obtained from an antecubital vein of the forearm by a trained member of the research team upon arrival to the laboratory for the assessment of plasma nitrate and nitrite. Samples were drawn into 6 mL lithium heparin tubes (Vacutainer, Becton–Dickinson, New Jersey, USA) and centrifuged at 3100 × *g* at 4 °C for 10 min within 2 min of collection. Plasma was extracted and stored in a − 80 °C freezer for the later analysis of plasma nitrate and nitrite using gas phase chemiluminescence as previously described (Hogwood et al. 2024a, b). Plasma nitrate and nitrite were assessed via ozone-based chemiluminescence using a Sievers NOA Model 280i (GE Analytical Instruments, Boulder, CO). Briefly, plasma nitrite of the undiluted plasma samples was determined by its reduction to nitric oxide in the presence of glacial acetic acid and potassium iodide. For plasma nitrate analysis, the plasma samples were deproteinized using cold ethanol precipitation in a 1:3 dilution (plasma:ethanol) before being centrifuged at 14,000 g for 10 min. The supernatant was removed for the subsequent plasma nitrate analysis in the presence of vanadium chloride in hydrochloric acid at 95 °C. For nitrate of the supplement conditions, 100 × and 150 × dilutions in deionized water were completed, and for nitrite, undiluted samples were injected. Plasma nitrite and nitrate were determined using the area under the curve in OriginPro software (v. 7.5 OriginLab, Northampton, MA).

#### Mood

The Brunel Mood Scale (BRUMS) (Terry et al. 1999, 2003) is used to assess mood states in adult populations and was conducted prior to exercise as mood may have a mediating effect on resistance training performance (Beedie et al. 2000). Using the standard response timeframe of “How do you feel right now?”, 24 items representing six subscales (i.e., anger, confusion, depression, fatigue, tension, vigor; four items per subscale) were captured using a five-point Likert scale (i.e., 0 = not at all, 1 = a little, 2 = moderately, 3 = quite a bit, 4 = extremely). Respective items were summed so that each subscale score ranged from 0 to 16 raw points. Elevated vigor and decreased anger, confusion, depression, fatigue, and tension subscale scores are viewed as positive outcomes.

#### Back squat and bench press performance

Power and velocity measurements were obtained during back squats and bench press using a portable, wireless, commercially available, linear position transducer (GymAware, Kinetic Performance Technology, Mitchell, Australia), which has been previously used (Tan et al. 2022, 2023c, 2025; Williams et al. 2020) and validated for test–retest reliability (Ballmann et al. 2021; Orange et al. 2020). During the 3 sets × 3 repetitions at 55%, 60%, and 65% 1RM, power and velocity were averaged across sets for the determination of mean power and mean velocity, and the highest power and velocity values were recorded for the determination of peak power and peak velocity.

#### Statistical analysis

A Student’s paired *t*-test was used to investigate differences across conditions (PL and BR) and exercise performance. Normality of paired differences was assessed using the Shapiro–Wilk test. A Bonferroni correction was applied for multiple comparisons using alpha of 0.05/number of comparisons. Effect sizes for *t*-tests were assessed using change scores with Hedge’s *g*_*z*_ correction applied, in which small, medium, and large effects were operationalized as 0.2, 0.5, and 0.8, respectively (Cohen 1988; Lakens 2013). Pearson product-moment correlation coefficients were used to assess the relationships between changes in plasma [nitrite] and performance variables, in which weak, moderate, and strong correlations were operationalized as 0.2, 0.5, and 0.8, respectively. Statistical significance was set to *P* ≤ 0.05 and all data are presented as mean ± standard deviation (SD) unless otherwise stated. All data was analyzed using SPSS version 27 (IBM, Armonk NY). Figures were generated using GraphPad Prism version 10.6.1.

Modelled individual change scores for all outcomes were calculated as the difference between the BR and PL conditions (BR − PL). Consistent with previous work, individual responses were then evaluated relative to the smallest worthwhile change (SWC), a threshold defined as 0.2 times the baseline between-individual standard deviation for each performance outcome during the PL condition (Swinton et al. 2018). Individual responses were classified as “meaningful”, “uncertain” or “adverse” if their observed changes lay above a 1 × SWC threshold, lay below a 1 × SWC threshold, or diminish beyond a 1 × SWC threshold (in the opposite direction of intended change), respectively.

### Strategies to reduce potential bias

To align with methodological best practices, we implemented rigorous procedures to control for various biases. This study was preregistered and the performance outcomes reported align with the registry. Computer-generated randomization and allocation concealment minimized selection bias. To mitigate performance bias, participants were blinded to the study hypothesis. The preparation, blinding and distribution of sensory-matched supplements to participants was performed by a researcher who was not formally involved in data collection. Data collection was performed by researchers who were blinded to experimental conditions for the entire duration of the experiment and who read from a standardized script for all visits. Data analysis was performed by researchers who were blinded to experimental conditions and who were not formally involved in data collection to reduce observer bias. The number of dropouts, the specific reasons for dropout, and the number of participants included in final analyses were reported to mitigate attrition bias.

## Results

All participants reported consuming all servings of each supplement at the correct times, that their menstrual cycle began and that they had maintained their habitual exercise and dietary habits prior to each testing visit via verbal confirmation and through a recorded questionnaire. There were no reported side effects from the supplement or perceived differences in taste between conditions. While the minimum washout period was set as 2 days minimum, all washout periods were > 2 days in duration to ensure experimental visits were performed during the early follicular phase.

### Plasma nitrate and nitrite

Plasma nitrate and nitrite are displayed in Table 1 for a subset of data (*n* = 15) owing to unsuccessful blood draws (*n* = 2) and sample contamination (*n* = 1). The coefficient of variation (CV%) for duplicate samples was 5.13%, for plasma nitrate. The placebo of nitrate-depleted beetroot juice contained negligible nitrate and the nitrate-rich of beetroot juice contained 6.7 mmol of nitrate. There was a significant difference between conditions in plasma nitrate (*P* < 0.001, *g*_*z*_ = 5.76) and nitrite (*P* < 0.001, *g*_*z*_ = 1.56). No significant associations were found between the change in plasma [nitrite] and any of the performance variables (Supplementary Table 1 and 2).

**Table 1:** Nitric oxide biomarkers following an acute dose of nitrate and placebo (*n* = 15)

Variable	PL	BR
Plasma Nitrate (µM)	37 ± 9	394 ± 56^*^
Plasma Nitrite (nM)	78 ± 33	394 ± 186^*^

*BR* nitrate-rich beetroot juice, *PL* nitrate-depleted beetroot juice

*Significant difference compared to PL (*P* < 0.001);

### Mood

There was no effect of condition on the six subcategories of mood (Table 2): anger (*P* = 0.21; *g*_*z*_ = 0.30), confusion (*P* = 0.16; *g*_*z*_ = 0.33), depression (*P* = 0.54; *g*_*z*_ = 0.14), fatigue (*P* = 0.73; *g*_*z*_ = 0.08), tension (*P* = 1.00; *g*_*z*_ = 0.00), vigour (*P* = 0.68; *g*_*z*_ = -0.10) before and after Bonferroni corrections.

**Table 2:** Summary of variations in mood across all experimental visits

	Placebo		Nitrate	
Variable	Average	Median	Average	Median
Anger	0.33 ± 0.84	0.00	0.06 ± 0.24	0.00
Confusion	0.11 ± 0.32	0.00	0.00 ± 0.00	0.00
Depression	0.33 ± 0.69	0.00	0.22 ± 0.65	0.00
Fatigue	2.50 ± 2.04	2.00	2.33 ± 1.64	2.00
Tension	0.67 ± 1.03	0.00	0.67 ± 1.08	0.00
Vigor	5.06 ± 3.54	5.00	5.33 ± 2.87	5.50

### Back squat performance

Back squat 1RM, 55%1RM, 60%1RM, and 65%1RM was 80 ± 18 kg, 44 ± 10 kg, 48 ± 10 kg, and 52 ± 12 kg, respectively. Performance outcomes during explosive barbell back squats are displayed in Table 3. There was no effect of condition on back squat peak power at 55%1RM (*P* = 0.66; *g*_*z*_ = 0.10), 60%1RM (*P* = 0.63; *g*_*z*_ = -0.11), 65%1RM (*P* = 0.68; *g*_*z*_ = -0.10), mean power at 55%1RM (*P* = 0.21; *g*_*z*_ = 0.29), 60%1RM (*P* = 0.99; *g*_*z*_ = 0.00), 65%1RM (*P* = 0.31; *g*_*z*_ = 0.24), peak velocity at 55%1RM (*P* = 0.88; *g*_*z*_ = 0.04), 60%1RM (*P* = 0.80; *g*_*z*_ = -0.06), 65%1RM (*P* = 0.27; *g*_*z*_ = -0.25) and mean velocity at 55%1RM (*P* = 0.30; *g*_*z*_ = 0.27), 60%1RM (*P* = 0.83; *g*_*z*_ = 0.06), 65%1RM (*P* = 0.39; *g*_*z*_ = 0.14) before and after Bonferroni corrections. Individual changes, patterns of response and rates of response for all performance outcomes are presented in Supplementary Material.

**Table 3:** 55%1RM, 60%1RM, and 65%1RM back squat performance following an acute dose of nitrate and placebo

Variable		Placebo	Nitrate	% Difference
Peak Power (W)	55%1RM	718 ± 212	725 ± 218	0.97
	60%1RM	761 ± 207	753 ± 215	− 1.05
	65%1RM	827 ± 219	820 ± 234	− 0.85
Mean Power (W)	55%1RM	319 ± 80	324 ± 79	1.57
	60%1RM	332 ± 81	332 ± 81	0.00
	65%1RM	333 ± 86	341 ± 81	2.40
Peak Velocity (m/s)	55%1RM	1.35 ± 0.13	1.35 ± 0.14	0.00
	60%1RM	1.30 ± 0.12	1.29 ± 0.14	− 0.77
	65%1RM	1.30 ± 0.12	1.28 ± 0.13	− 1.54
Mean Velocity (m/s)	55%1RM	0.74 ± 0.08	0.76 ± 0.06	2.70
	60%1RM	0.71 ± 0.08	0.71 ± 0.07	0.00
	65%1RM	0.67 ± 0.09	0.68 ± 0.07	1.49

*1RM* one-repetition maximum; m/s = meters per second, *W* watts; % Difference = [(Nitrate-Placebo)/Placebo]*100

### Bench press performance

**Fig. 1:**
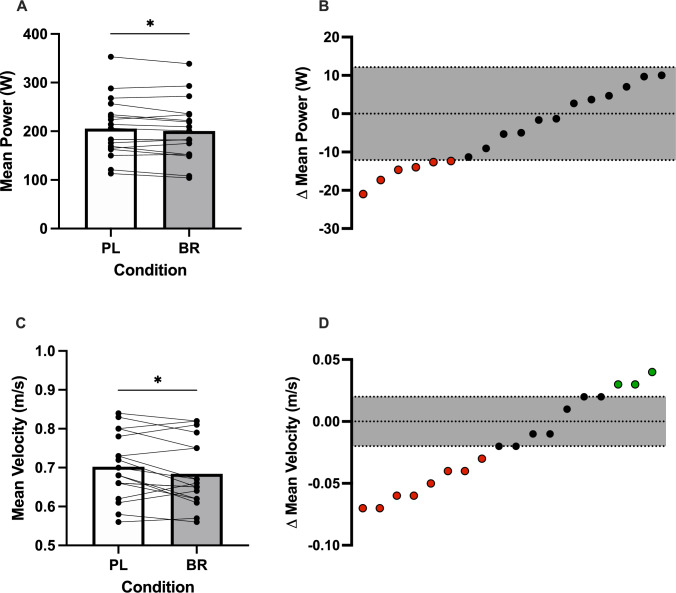
Mean, individual and exploratory analysis of smallest worthwhile change (SWC) for power and velocity during 60% 1RM barbell bench press after consuming a single dose of nitrate-depleted placebo (PL) and nitrate-rich beetroot juice (BR). **A** Group mean power and (**B**) individual power responses; **C** group mean velocity and (**D**) individual velocity responses. For exploratory analysis (**B** and **D**), coloured data points reflect individual response classifications relative to the SWC; green points represent a “meaningful” response (> 1 × SWC); black data points captured within the shaded region represent an “uncertain” response (± 1 × SWC); red data points represent an “adverse" response (< -1 × SWC). MPO = mean power output; MV = mean velocity; m/s = meters per second; W = Watts; 1RM = 1-repetition max. * = *P* ≤ 0.05

Bench press 1RM, 55%1RM, 60%1RM, and 65%1RM was 50 ± 14 kg, 28 ± 10 kg, 31 ± 11 kg, and 34 ± 13 kg, respectively. Performance outcomes during barbell bench press are displayed in Table 4. Bench press performance was significantly lower compared to PL for 60%1RM in mean power (*P* = 0.047; *g*_*z*_ = -0.48; Fig. 1) and mean velocity during 60%1RM (*P* = 0.049; *g*_*z*_ = -0.50; Fig. 1) before Bonferroni corrections; however, there was no significance after Bonferroni corrections were applied. There was no effect of condition on bench press peak power at 55%1RM (*P* = 0.50; *g*_*z*_ = -0.15), 60%1RM (*P* = 0.60; *g*_*z*_ = -0.12), 65%1RM (*P* = 0.28; *g*_*z*_ = -0.25), mean power at 55%1RM (*P* = 0.48; *g*_*z*_ = -0.16), 65%1RM (*P* = 0.23; *g*_*z*_ = -0.28), peak velocity at 55%1RM (*P* = 0.30; *g*_*z*_ = -0.24), 60%1RM (*P* = 0.20; *g*_*z*_ = -0.30), 65%1RM (*P* = 0.97; *g*_*z*_ = -0.01) and mean velocity at 55%1RM (*P* = 0.18; *g*_*z*_ = -0.33), 65%1RM (*P* = 0.33; *g*_*z*_ = -0.26) before and after Bonferroni corrections. Individual changes, patterns of response and rates of response for all performance outcomes are presented in Supplementary Material.

**Table 4:** 55%1RM, 60%1RM, and 65%1RM bench press performance following an acute dose of nitrate and placebo

Variable		Placebo	Nitrate	% difference
Peak Power (W)	55%1RM	337 ± 103	331 ± 102	− 1.78
	60%1RM	326 ± 96	324 ± 105	− 0.61
	65%1RM	332 ± 99	321 ± 97	− 3.31
Mean Power (W)	55%1RM	197 ± 56	193 ± 54	− 2.03
	60%1RM	206 ± 61	201 ± 61*	− 2.43
	65%1RM	202 ± 60	197 ± 56	− 2.48
Peak Velocity (m/s)	55%1RM	1.12 ± 0.13	1.10 ± 0.16	− 1.79
	60%1RM	1.01 ± 0.13	1.00 ± 0.15	− 0.99
	65%1RM	0.92 ± 0.13	0.92 ± 0.13	0.00
Mean Velocity (m/s)	55%1RM	0.74 ± 0.09	0.72 ± 0.09	− 2.70
	60%1RM	0.70 ± 0.08	0.68 ± 0.08*	− 2.86
	65%1RM	0.64 ± 0.09	0.63 ± 0.07	− 1.56

*1RM* one-repetition maximum, *m/s* meters per second, *W* watts, *% Difference* [(Nitrate-Placebo)/Placebo]*100

*P ≤ 0.05

## Discussion

The main novel finding of the present study was that there were no beneficial effects of dietary nitrate on upper- and lower-body resistance exercise performance in resistance-trained women. Additionally, mean power (-2.4%) and mean velocity (-3%) were lowered in 60%1RM barbell bench press (P < 0.05) following an acute nitrate dose (6.7 mmol nitrate) in resistance-trained women although these effects were not present after the Bonferonni correction was applied. These findings conflict with our hypothesis and suggest that dietary nitrate supplementation has no effects on barbell back squat performance and most of the bench press performance indices assessed, but may compromise some aspects of bench press performance in resistance-trained women. These data do not support dietary nitrate as an effective strategy to improve explosive weightlifting performance in resistance-trained women and further research is required to elucidate whether dietary nitrate has ergolytic effects in this context. 

### No ergogenic effects of dietary nitrate on resistance exercise performance in women

Far fewer studies have assessed the potential for dietary nitrate supplementation to improve exercise performance in women-only cohorts compared to men (Meng et al. 2025; Senefeld et al. 2020), with these women-only studies having reported equivocal effects (Buck et al. 2015; López-Samanes et al. 2022a, b, , 2023; Tan et al. 2024; Wickham et al. 2019). Notably, some studies have found that short-term (3–5 days) moderate nitrate dosing (~ 13 mmol per day) worsened severe-intensity cycling time-to-exhaustion performance (Hogwood et al. 2023) and knee extension power and velocity (Hogwood et al. 2024a, b). Our original findings extend these observations by showing that an acute low nitrate dose (6.7 mmol nitrate) compromised some aspects, but not all aspects, of explosive bench press performance, and did not impact power and velocity during back squats in resistance-trained women.

Previous studies found that greater elevations in plasma [nitrite] (Coggan et al. 2018; Porcelli et al. 2015; Wilkerson et al. 2012) and muscle [nitrate] (Kadach et al. 2023) following dietary nitrate supplementation increased the likelihood of ergogenic effects in cycling, running and knee extensions in healthy men. In the present study, we did not observe significant correlations between plasma [nitrite] and any weightlifting performance outcomes. However, recent studies from our laboratory and others have provided additional insight as it was observed that a greater increase in plasma [nitrite], and thus, potential for greater nitric oxide synthesis, was associated with less beneficial effects of nitrate in barbell back squats (Tan et al. 2025) and isokinetic knee extensions (Gallardo et al. 2021; Wei et al. 2025). While these data suggest that a lower dose might increase efficacy, the current study provided 6.7 mmol of nitrate––which aligns with the currently accepted minimum nitrate dose for eliciting beneficial effects (> 5 mmol nitrate or 1 nitrate-rich beetroot shot containing ~ 6–6.5 mmol nitrate (Senefeld et al. 2020))––but did not find any positive effects. Importantly, this dose has been established for endurance and repeated sprinting exercise (Senefeld et al. 2020), but the optimal dose for resistance exercise and weightlifting performance enhancement (Tan et al. 2025), and particularly in women, is unclear and requires more research. Current dosing guidelines are based on only one early study conducted on cycling performance and in healthy Caucasian men from the United Kingdom which limits its application to women and other exercise modalities and populations (Wylie et al. 2013).

We observed that acute dietary nitrate ingestion appeared to worsen mean power and mean velocity during 60%1RM barbell bench press (*P* < 0.05); however, the significant effects were not present after applying the Bonferroni method. Additionally, there were no effects of dietary nitrate during 55%1RM and 65%1RM barbell bench press. These data indicate the potential for dietary nitrate to negatively impact some aspects of bench press performance and that mean power and mean velocity at 60%1RM may be worth additional focus in future studies to verify these findings. Only 9 studies have examined the effects of dietary nitrate on neuromuscular performance (i.e., power and velocity) during weightlifting exercise, in which 5 studies reported improved power (+ 6–22%) and/or velocity (+ 6–28%) (Jurado-Castro et al. 2022; Rodríguez-Fernández et al. 2021; Salem et al. 2025; Williams et al. 2020) while 4 studies did not observe any effects (Montalvo-Alonso et al. 2025; Tan et al. 2022, 2023c, 2025). To the authors’ knowledge, this is the first study to investigate nitrate supplementation on bench press performance in women.

We did not observe any influence of dietary nitrate supplementation on 55%1RM, 60%1RM, and 65%1RM barbell back squats. These results are in agreement with some (Montalvo-Alonso et al. 2025; Ranchal-Sanchez et al. 2020; Salem et al. 2025; Tan et al. 2022, 2023c, 2025) but not all studies (Jurado-Castro et al. 2022; Rodríguez-Fernández et al. 2021). Importantly, apart from the present study, only Jurado-Castro et al. examined effects of dietary nitrate on back squat performance in a women-only cohort (Jurado-Castro et al. 2022). Contrary to the present study, Jurado-Castro et al. found that an acute low nitrate dose (~ 6 mmol nitrate) improved mean velocity (+ 6.7%), peak velocity (+ 6%), mean power (+ 7.3%), and peak power (+ 6.8%) during Smith-machine back squats at 50%1RM but not 75%1RM in recreationally active women (Jurado-Castro et al. 2022).

The discrepancy in performance effects between the present study and Jurado-Castro et al. could be due to differences in: (1) exercise protocol, modalities, and best practices for weightlifting; (2) standardization of tempo, instructions, and cues; (3) equipment for weightlifting and performance outcomes; (4); population (e.g., definition of “resistance-trained”, and fiber-type composition); and (5) controls for placebo, lifestyle habits, time of day, and menstrual cycle (Haff & Triplett, 2016; Jurado-Castro et al. 2022; Tan et al. 2023b; Weakley et al. 2021). Comprehensive and rigorous methodological designs appear important in assessing dietary nitrate and weightlifting performance since studies with better control measures have not observed positive effects while others yielded large effects (Tan et al. 2023b). Additionally, it is unclear why some bench press outcomes were potentially compromised; however, it has been speculated that nitric oxide balance is important for the efficacy of nitrate on performance in women and is more complex than merely increasing circulating nitrite (Forte et al. 1998; Hogwood et al. 2023).

The classification of “resistance-trained” can encompass a broad range of experience levels, from recreationally active individuals to highly trained athletes; therefore, training age and elite-level weightlifting experience may be important factors to consider that could modulate the ergogenic potential of dietary nitrate supplementation. Similarly, some recent data have suggested that dietary nitrate supplementation may be less likely to be ergogenic in women (Hogwood et al. 2023) which is in contrast to studies conducted in men (Senefeld et al. 2020) highlighting the importance of potential sex differences. No studies to date have examined the effects of dietary nitrate in resistance-trained women at the elite level. Although dietary nitrate literature frequently suggests that highly trained or elite athletes are less likely to gain an ergogenic benefit from nitrate supplementation, a key nuance is that the available evidence primarily indicates reduced efficacy in individuals with high aerobic capacity (> 65 ml/kg/min) (e.g., Porcelli et al. 2015; Wilkerson et al. 2012). Extending this finding to all elite athletes is potentially misleading as elite sport is characterized by more than high aerobic capacity and includes athletes with diverse metabolic profiles and phenotypes. Consequently, dietary nitrate may still have potential efficacy in elite female athletes with high training ages in weightlifting, such as in power- and strength-based disciplines, given that nitrate may preferentially influence type II muscle fiber function (Ferguson et al. 2013; Hernández et al. 2012) and that skeletal muscle mass appears to have a role (Kadach et al. 2023). Additional research is required to clarify the effects of dietary nitrate across different elite athletic populations and in women(Tan et al. 2023a).

### Individual responses

Quantifying individual responses and meaningful change is rare in exercise science research, but various methods are available including gold standard repeatability crossover trials (Hayes et al. 2025); calculating minimum clinically important difference of extensively studied outcomes (Margaritelis et al. 2023) or smallest worthwhile change (SWC) (Renwick et al. 2025); and more simplistically and less rigorously, the “responder vs. non-responder” dichotomies (Wylie et al. 2013). We implemented SWC to define “meaningful change” for power and velocity during barbell back squats and bench press since there are no universally accepted “clinically meaningful” thresholds for these metrics and exercise modality. We defined the response threshold for power and velocity based on the baseline between-individual standard deviation for each performance outcome (Bonafiglia et al. 2018; Swinton et al. 2018). This exploratory analysis provides insight beyond the *P* value and mean data; our SWC threshold revealed substantial heterogeneity across all performance outcomes across exercise intensities, modalities, and within and across participants (Supplementary Materials). These data reveal that there are no global “responders” or “non-responders”, and rather, that the majority of responses are uncertain and highly variable within and between participants. While the SWC statistical framework is imperfect, the highly variable results observed in our exploratory analysis serve as an impetus for further consideration and investigation of individual responses to dietary nitrate supplementation. Additionally, caution is warranted to avoid overinterpretation of these findings. Exploring intra- and inter-individual variability is particularly relevant in applied sporting contexts where even marginal performance changes can determine outcomes (e.g., hundredths to a few tenths of a second can shift podium outcomes from finalist to a medalist at the Olympic level (Turner et al. 2025)). However, based on the present findings, inorganic dietary nitrate does not appear to be ergogenic for weightlifting performance in women.

### Limitations and future directions

We acknowledge that adequately characterizing individual variability requires larger data sets and gold standard study designs where possible, and thus, our exploratory analysis of the SWC is intended only to highlight intra- and inter-individual variability as an important consideration for future research. We did not directly measure hormonal profiles to confirm that participants were tested on days 1–5 of menses (i.e., early follicular phase) or quantify symptoms associated with menses. However, our study did not aim to draw conclusions specific to the early follicular phase; rather, we used this time frame to control for hormonal variability within and across participants which our study design accomplishes (Baranauskas et al. 2022; Elliott-Sale et al. 2025). Future studies should investigate, for example, how weightlifting protocols/modalities, performance outcomes, supplementation strategies, nitric oxide biomarkers, population/training status, repeatability crossover trials, direct sex comparisons etc., impact potential ergogenic effects of nitrate supplementation on weightlifting performance in women, as well as how findings translate from strength training centers to practical and meaningful changes in sport-specific contexts.

## Conclusion

An acute nitrate dose did not improve any performance outcome in barbell back squats and barbell bench press at 55%1RM, 60%1RM, and 65%1RM in resistance-trained women. Thus, while dietary nitrate supplementation may confer performance-enhancing effects on cycling and running in men, as evidenced by the myriads of studies conducted in men-only cohorts, our results suggest that its effects may not be beneficial in women, at least for explosive weightlifting performance. More research is required to verify if dietary nitrate is detrimental to weightlifting performance in women.

## Supplementary Information

Below is the link to the electronic supplementary material.

Supplementary file1 (DOCX 181 KB)

## Data Availability

The experimental data and the simulation results that support the findings of this study are available upon request.
