# Supplementary material for: Dietary nitrate supplementation does not improve resistance exercise performance in resistance-trained women
Source: Eur J Appl Physiol. 2026 Apr 3;126(7):3677–89. doi: 10.1007/s00421-026-06206-9 (PMC13380560; doi:10.1007/s00421-026-06206-9)
Supplement: Supplementary file 1 — Supplementary file1 (DOCX 181 KB) [file 421_2026_6206_MOESM1_ESM.docx]

**Supplementary Materials**

**Supplementary Figure 1.** Individual responses for performance outcomes at 55%1RM, 60%1RM and 65%1RM barbell bench press in relation to the corresponding smallest worthwhile change. 1RM = one-repetition maximum. MPO = mean power output; MV = mean velocity; PPO = peak power output; PV = peak velocity; Resp = % of individuals demonstrating a meaningful response vs. total sample size.

**Supplementary Figure 2.** Individual responses for performance outcomes at 55%1RM, 60%1RM and 65%1RM barbell back squat in relation to the corresponding smallest worthwhile change. 1RM = one-repetition maximum. MPO = mean power output; MV = mean velocity; PPO = peak power output; PV = peak velocity; Resp = % of individuals demonstrating a meaningful response vs. total sample size.

| **Supplementary Table 1.** Correlation analyses comparing the change in plasma nitrite concentrations and the change in performance outcomes during back squats. | | | |
| --- | --- | --- | --- |
| **Variable** | | ***r*** | ***P*-value** |
| ΔPeak Power (W) | 55%1RM | 0.007 | 0.978 |
|  | 60%1RM | 0.189 | 0.451 |
|  | 65%1RM | -0.223 | 0.375 |
| ΔMean Power (W) | 55%1RM | -0.126 | 0.618 |
|  | 60%1RM | -0.176 | 0.484 |
|  | 65%1RM | 0.301 | 0.225 |
| ΔPeak Velocity (m/s) | 55%1RM | 0.305 | 0.218 |
|  | 60%1RM | 0.269 | 0.281 |
|  | 65%1RM | 0.068 | 0.790 |
| ΔMean Velocity (m/s) | 55%1RM | -0.289 | 0.245 |
|  | 60%1RM | -0.369 | 0.131 |
|  | 65%1RM | -0.244 | 0.329 |
| 1RM = one-repetition maximum; m/s = meters per second; *r* = Pearson correlation coefficient; W = watts; Δ = (PL-BR) | | | |

| **Supplementary Table 2.** Correlation analyses comparing the change in plasma nitrite concentrations and the change in performance outcomes during bench press. | | | |
| --- | --- | --- | --- |
| **Variable** | | ***r*** | ***P*-value** |
| ΔPeak Power (W) | 55%1RM | -0.123 | 0.626 |
|  | 60%1RM | -0.028 | 0.914 |
|  | 65%1RM | -0.075 | 0.767 |
| ΔMean Power (W) | 55%1RM | -0.254 | 0.309 |
|  | 60%1RM | -0.015 | 0.951 |
|  | 65%1RM | -0.162 | 0.521 |
| ΔPeak Velocity (m/s) | 55%1RM | 0.186 | 0.461 |
|  | 60%1RM | 0.185 | 0.462 |
|  | 65%1RM | -0.135 | 0.594 |
| ΔMean Velocity (m/s) | 55%1RM | -0.253 | 0.311 |
|  | 60%1RM | -0.034 | 0.892 |
|  | 65%1RM | -0.079 | 0.756 |
| 1RM = one-repetition maximum; m/s = meters per second; *r* = Pearson correlation coefficient; W = watts; Δ = (PL-BR) | | | |
